# Bone turnover markers in patients with inflammatory bowel disease in remission: a cross-sectional comparison of anti-TNFα therapy with conventional maintenance therapy

**DOI:** 10.3389/fmed.2026.1729584

**Published:** 2026-02-10

**Authors:** S. Jarmakiewicz-Czaja, P. R. Kiela, D. Piątek, A. Sokal-Dembowska, W. Guz, R. Radzki, J. Sztembis, R. Filip

**Affiliations:** 1 Faculty of Health Sciences and Psychology, Collegium Medicum, University of Rzeszów, Rzeszów, Poland; 2 University Center for Research and Development in Health Sciences, Rzeszów, Poland; 3 Department of Pediatrics, Daniel Cracchiolo Institute for Pediatric Autoimmune Disease Research, Steele Children's Research Center, University of Arizona, Tucson, AZ, United States; 4 Chair and Department of Conservative Dentistry with Endodontics, Medical University of Lublin, Lublin, Poland; 5 Department of Electroradiology, Medical College, University of Rzeszow, Rzeszow, Poland; 6 Department of Radiology, St. Jadwiga Queen Hospital No. 2, Rzeszow, Poland; 7 Department of Animal Physiology, Faculty of Veterinary Medicine, University of Life Sciences in Lublin, Poland; 8 Endocrinology Clinic, Pro-Familia Specialist Hospital, Rzeszów, Poland; 9 Department of Gastroenterology with IBD Unit, St. Jadwiga Queen Hospital No. 2 Affiliated to the Medical College, University of Rzeszow, Rzeszow, Poland; 10 Department of Internal Medicine, Medical College, University of Rzeszow, Rzeszow, Poland

**Keywords:** Crohn's disease, inflammatory bowel disease, osteoporosis, TNFα, ulcerative colitis

## Abstract

**Introduction:**

Inflammatory bowel diseases (IBD), including Crohn's disease (CD) and ulcerative colitis (UC), are associated with increased risk of bone loss due to chronic inflammation, nutritional deficiencies, and pharmacological treatment.

**Purpose:**

The purpose of this study was to evaluate bone mineral density (BMD) and selected bone turnover markers in IBD patients in clinical and endoscopic remission, treated with conventional therapy or anti-TNF-α agents.

**Methods:**

This single-center study included 100 participants: 35 with CD, 37 with UC, and 28 age-matched healthy controls. Patients IBD participated in the study received conventional treatment or anti- TNF-α therapy with ADA (adalimumab) or IFX (infliximab). IBD patients were in confirmed remission, without steroid use or comorbidities affecting bone metabolism. BMD was measured using dual-energy X-ray absorptiometry (DXA) at the lumbar spine (L2–L4), the left femoral neck, and whole body. Serum levels of osteocalcin (OC), parathyroid hormone (PTH), sclerostin (SOST), fibroblast growth factor 23 (FGF23), Dickkopf-1 (DKK1), osteoprotegerin (OPG), and osteopontin (OPN) were assessed.

**Results:**

The IBD group demonstrated a statistically significant higher OPN value (*p* < 0.001) compared to the control group. Additionally, the total T-score revealed a significant difference between the groups (*p* = 0.005), with the control group exhibiting the highest values. No significant differences were found in the levels of other bone density markers studied between the biologically treated group and the conventionally treated group.

**Conclusions:**

Our study indicates that patients with IBD are at risk of developing reduced bone mineral density and osteoporosis. While some bone turnover markers appear to normalize during remission, anti-TNF-α treatment does not offer added benefits for bone metabolism compared to conventional therapy.

## 1 Introduction

Inflammatory bowel diseases (IBD) include Crohn's disease (CD), ulcerative colitis (UC) and microscopic inflammatory bowel disease. They go through periods of remission and exacerbation. In CD, inflammation can be localized in any part of the gastrointestinal tract, from the mouth, through the esophagus, stomach, small intestine, large intestine, and rectum. Inflammation can affect the entire thickness of the gastrointestinal wall. In UC, inflammation usually begins in the rectum and spreads proximally to the large intestine. The nature of inflammatory changes is continuous and involves the mucosa and submucosa (1, 2).

IBDs are characterized by recurrent immune disorders involving both anti-inflammatory and pro-inflammatory factors. An abnormal inflammatory response can lead to a range of clinical manifestations (3). The goal of treatment is to achieve and maintain deep remission (healing of the intestinal mucosa), thus reducing the risk of the need for surgical treatment. When considering the implementation of appropriate treatment, the activity and severity of the disease course, the response to previously used medications, and concomitant extraintestinal symptoms, among other factors, should be taken into account. Treatment modalities can be divided into pharmacological and surgical. An adjunctive component of primary treatment is nutritional therapy (4). The most common pharmacological treatment is based on traditional drugs (5-ASA, Sulfasalazine) and biological treatment based on the use of anti-TNF-α drugs (3).

The prevalence of low bone density in patients with CD and UC is about 42% (5). IBD has a higher risk of reduced bone density than the general population. This is due in part to the prolonged active phase of the disease. During the occurrence of inflammation, cytokines are produced that enhance bone resorption (increase RANKL—RANK receptor ligand to OPG—osteoprotegerin). During prolonged remission of the disease, patients may increase bone mineral density (BMD) (6). Patients with IBD often have progressive clinical symptoms due to worsening nutritional deficiencies, the treatment used, the inflammation present, or a genetic predisposition (7, 8). After achieving remission in patients with IBD, without other risk factors for osteoporosis, bone turnover markers should return to normal. However, whether this is also the case in patients treated with TNF-alpha blocking drugs remains an open question. It is known that in terms of preventing bone loss, anti-TNF drugs do not have an advantage over traditional IBD treatment (9, 10). However, more recent data indicate that anti-TNFα therapy is associated with increased bone turnover markers and decreased BMD in the femoral neck (11).

The aim of the study was to determine whether bone mineral density and selected regulatory molecules [OC (osteocalcin), PTH (parathyroid hormone), SOST (sclerostin), FGF23 (fibroblast growth factor 23), DKK1 (Dickkopf-related protein 1), OPG, OPN (osteopontin)] in patients with IBD during remission maintenance treatment are normalized or remain outside normal limits.

## 2 Material and methods

### 2.1 Study population

This was a single-center study conducted among patients with CD and UC. Patients recruited for the study received conventional treatment or anti- TNF-α therapy with ADA (adalimumab) or IFX (infliximab; without hormone therapy). It was carried out in the tertiary IBD center in Rzeszow (Poland), according to the Good Clinical Practice Guidelines, the Declaration of Helsinki principles, and was approved by the local Ethics Committee (KE-0254/68/2015 and 23/04/2016).

A total of 100 subjects were recruited for the study, including 35 subjects with a diagnosis of CD, 37 subjects with a diagnosis of UC, and 28 healthy volunteers who were in a suitable age-matched group for the study. Patients with IBD were in remission of the disease.

The main inclusion criteria for the study were: IBD diagnosis CD or UC at least 2 years before entry to the study remission phase of the disease, standard treatment with 5-aminosalicylic or a standard dose of an immunosuppressive drug (e.g., Azathioprine 1 mg/kg) or IFX or ADA.

The exclusion criteria for the study were: current steroid use, active phase of the disease, pregnancy, with chronic comorbidities or therapy that could affect bone turnover marker levels.

We verify the diagnosis of IBD according to the 2018 recommendations of the American College of Gastroenterology (12, 13) and the European Organization for Crohn's and Colitis (14, 15).

### 2.2 Measurement of bone density

The bone mineral density was assessed using dual-energy x-ray absorptiometry (DXA). The study was conducted at the University of Rzeszów using a Lunar iDXA densitometer from GE Healthcare Lunar (Madison, Wisconsin, USA), which was equipped with enCORE software. Bone mineral density was measured in the lumbar spine (L2–L4 area), the left femoral neck, and the entire body. The estimated precision errors for the above scan areas are 0.010 g/cm2 (1%) (16). The DXA scan was performed on patients on average 7.1 years after diagnosis (Me = 7 years, SD ± 4.37, *n* = 54).

### 2.3 Measurement of bone turnover markers

Bone turnover markers in serum samples were measured using multiplex MILLIPLEX^®^ Human Bone Magnetic Bead Panel (Millipore Sigma; St. Louis, MO) and MagPix (Diasorin; Austin, TX) instrument. According to the manufacturer, intra-assay CV was < 10% and inter-assay CV < 15%, with no cross-reactivity observed between analytes. Curve fitting and initial data analysis were performed with Belysa Immunoassay Curve Fitting Software (Millipore Sigma).

### 2.4 Laboratory assessments

All laboratory tests (hsCRP, calprotectin, blood morphology) were performed using routine commercial tests. CRP was measured using the CRP4 Tina-quant C-Reactive Protein IV CRP (Roche Diagnostics GmbH, Mannheim, Germany). Detection limit 0.1–0.22 mg/L, sensitivity 0.042 mg/dL (0.42 mg/L), while calprotectin was measured using the Bühlmann fCAL turbo assay (Bühlmann Diagnostics, Schönenbuch, Switzerland; measuring range 20–8000 μg/g).

### 2.5 Statistical analysis

All results are expressed as median, standard deviation. The Kruskal-Wallis test was used, followed by Dunn's *post-hoc* test with Bonferroni correction. For normally distributed variables, ANOVA with Tukey's HSD test was used, which automatically corrects the results for multiple comparisons. In the case of correlations, Spearman's rank correlation was used due to the skewed distribution of biochemical markers. All analyses were performed using Statistica version 13 computer statistical software licensed from the University of Rzeszow. A level of *p* < 0.05 was considered the level of statistical significance.

## 3 Results

There were no significant differences between the groups (CD, UC and the control group) in terms of age, height, weight, body mass index (BMI) and, basic laboratory assessments like hemoglobin, high sensitivity C-reactive protein (hs-CRP) and calprotectin. The mean time to disease diagnosis was comparable between CD and UC (Supplementary material).

All patients had no clinical symptoms of CD or UC followed by normal values of inflammatory markers, which confirmed remission of the disease. Moreover, within the last 6 months, all patients passed endoscopic examination which also confirmed endoscopic remission. The criteria for CD remission were a simple Endoscopic Score for Crohn's Disease (SES-CD) of no greater than 4, with at least a 2-point reduction compared to the baseline of induction and no subscore greater than 1 (17), and for UC as a Mayo endoscopy Subscore (MES) (18).

A detailed *post-hoc* analysis (Dunne's test with Bonferroni correction) revealed different patterns of significance for the markers studied. OPN values were significantly higher in the CD and UC groups compared to the control group (*p* < 0.001). In addition, statistically significant differences between groups were observed in OC levels (*p* = 0.028). The other markers (DKK1, OPG, SOST, PTH, and FGF23) did not show statistically significant differences (Table 1).

**Table 1:** Serum levels of bone turnover markers in the studied groups.

**Variables**	**CD group**			**UC group**			**Control group**			***p*-value**
	**N**	**Me**	**IQR**	**N**	**Me**	**IQR**	**N**	**Me**	**IQR**	
DKK1 (pg/mL)	35	4,303.72	3,099.02	37	4,798.27	2,627.51	27	4,222.69	1,995.19	0.362
OPG (pg/mL)	35	561.08	244.14	37	666.59	327.71	27	604.11	192.00	0.272
OC (pg/mL)	35	21,318.54	30,557.49	37	21,018.84	26,565.15	27	43,977.93	73,437.61	**0.028**
OPN (pg/mL)	35	19,345.59	11,439.38	37	15,735.60	20,380.77	28	7,951.33	5,727.69	**< 0.001**
SOST (pg/mL)	19	817.95	1,795.60	23	1,726.65	1,492.10	19	968.05	1,182.75	0.147
PTH (pg/mL)	34	88.80	70.05	37	97.53	67.33	27	104.98	59.80	0.800
FGF23 (pg/mL)	22	64.72	29.43	32	69.68	21.98	28	71.38	19.00	0.083

DKK1, Dickkopf-related protein 1; OPG, osteoprotegerin; OC, osteocalcin, OPN, osteopontin, SOST, sclerostin; PTH, parathyroid hormone; FGF23, fibroblast growth factor 23; CD, Crohn's disease; UC, ulcerative colitis; Me, median; IQR, Interquartile range.

There were no significant differences in L2–L4 BMD, L2–L4 T-score, L2–L4 Z-score, Total Z-score, and BMC values between all study groups. Only the total T-score showed a significant difference between the groups (*p* = 0.005), with the highest values in the control group (Table 2).

**Table 2:** Differences between groups in bone mineral density and bone mineral content values.

**Variables**	**CD**			**UC**			**Control group**			***p*-value**
	**N**	**Mean**	**SD**	**N**	**Mean**	**SD**	**N**	**Mean**	**SD**	
L2–L4 BMD	35	1.169	0.161	37	1.182	0.160	28	1.227	0.153	0.337
L2–L4 T-score	35	−0.260	1.327	37	−0.149	1.328	28	0.225	1.280	0.326
L2–L4 Z-score	35	−0.271	1.308	37	−0.186	1.316	28	0.007	1.178	0.684
Total T-score	35	0.246	1.173	37	0.451	1.050	28	1.146	1.046	**0.005**
Total Z-score	35	0.443	1.098	37	0.551	1.019	28	0.964	0.954	0.121
BMC	35	2,660.000	510.947	37	2,606.946	487.456	28	2,620.321	505.018	0.899

CD, Crohn's disease; UC, ulcerative colitis; SD, standard deviation; BMC, bone mineral content; BMD, bone mineral density.

Analysis of the impact of treatment type (anti-TNFα vs. conventional therapy) on bone turnover markers showed a statistically significant difference only in the case of OC. Patients treated conventionally had significantly higher median OC concentrations compared to patients receiving biological treatment (*p* = 0.041). No significant differences were observed between the subgroups for the other biochemical markers (*p* > 0.05; Table 3).

**Table 3:** Comparison of serum bone turnover marker levels in patients receiving biological (anti-TNFα) vs. conventional treatment.

**Variables**	**anti-TNF**α **treatment**			**Conventional treatment**			***p*-value**
	**N**	**Me**	**IQR**	**N**	**Me**	**IQR**	
DKK1 (pg/mL)	55	4,503.75	2,814.67	17	4,339.54	3,172.58	0.750
OPG (pg/mL)	55	561.08	273.64	17	676.23	212.66	0.254
OC (pg/mL)	55	20,919.74	24,122.55	17	35,069.54	43,691.44	**0.041**
OPN (pg/mL)	55	18,947.26	19,147.71	17	17,727.19	13,909.93	0.624
SOST (pg/mL)	30	893.00	1,721.41	12	1,633.86	1,671.02	0.404
PTH (pg/mL)	54	84.42	77.44	17	97.53	39.93	0.952
FGF23 (pg/mL)	40	67.20	19.26	14	70.01	38.91	0.176

DKK1, Dickkopf-related protein 1; OPG, osteoprotegerin; OC, osteocalcin; OPN, osteopontin; SOST, sclerostin; PTH, parathyroid hormone; FGF23, fibroblast growth factor 23; Me, median; IQR, interquartile range; Statistical significance assessed using Mann-Whitney U test.

There were no differences in L2–L4 BMD, L2–L4 T-score, L2–L4 Z-score, Total T-score, Total Z-score, and BMC values between the biologically and conventionally treated groups (Table 4).

**Table 4:** Comparison of bone mineral density and bone mineral content in patients receiving biological (anti-TNFα) vs. conventional treatment.

**Variables**	**Anti-TNF**α **treatment**			**Conventional treatment**			***p*-value**
	**N**	**Mean**	**SD**	**N**	**Mean**	**SD**	
L2–L4 BMD	55	1.171	0.161	17	1.193	0.156	0.620
L2–L4 T-score	55	−0.251	1.339	17	−0.047	1.277	0.581
L2–L4 Z-score	55	−0.295	1.316	17	−0.012	1.276	0.438
Total T-score	55	0.311	1.138	17	0.482	1.025	0.581
Total Z-score	55	0.449	1.064	17	0.659	1.030	0.477
BMC	55	2,625.436	515.226	17	2,656.353	442.567	0.824

SD, standard deviation; BMC, bone mineral content; BMD, bone mineral density; Statistical significance assessed using independent samples t-test.

The only significant correlation in patients treated with anti-TNFα was a negative correlation between FGF23 concentration and BMC (rho = −0.36; *p* < 0.05). However, no significant correlations were found between the other bone markers (DKK1, OPG, OC, OPN, SOST, and PTH) and bone density indices in this subgroup. Within the biochemical markers themselves, a statistically significant positive correlation was observed between DKK1 protein and OPG (rho = 0.41; *p* < 0.05) and a significant negative correlation between SOST and OC (rho = −0.43; *p* < 0.05; Table 5).

**Table 5:** Correlation analysis of bone turnover markers and bone density indices in a group of patients receiving anti-TNFα treatment.

**Parameters**	**L2–L4 BMD**	**L2–L4 T-score**	**Total T-score**	**BMC**	**DKK1**	**OPG**	**OC**	**OPN**	**SOST**	**PTH**	**FGF23**
L2–L4 BMD	-	1.00^*^	0.68^*^	0.71^*^	−0.05	0.03	0.03	0.02	0.05	−0.09	−0.29
L2–L4 T-score	1.00^*^	-	0.67^*^	0.71^*^	−0.05	0.03	0.02	0.03	0.05	−0.09	−0.29
Total T-score	0.68^*^	0.67^*^	-	0.67^*^	−0.19	−0.07	0.13	−0.12	0.10	0.01	−0.23
BMC	0.71^*^	0.71^*^	0.67^*^	-	−0.17	−0.18	0.19	0.14	−0.06	0.04	−0.36^*^
DKK1	−0.05	−0.05	−0.19	−0.17	-	0.41^*^	−0.21	−0.05	−0.03	0.07	0.15
OPG	0.03	0.03	−0.07	−0.18	0.41^*^	-	0.04	−0.04	0.23	0.01	0.21
OC	0.03	0.02	0.13	0.19	−0.21	0.04	-	−0.23	−0.43^*^	0.01	−0.21
OPN	0.02	0.03	−0.12	0.14	−0.05	−0.04	−0.23	-	0.03	0.18	0.10
SOST	0.05	0.05	0.10	−0.06	−0.03	0.23	−0.43^*^	0.03	-	0.09	0.11
PTH	−0.09	−0.09	0.01	0.04	0.07	0.01	0.01	0.18	0.09	-	0.05
FGF23	−0.29	−0.29	−0.23	−0.36^*^	0.15	0.21	−0.21	0.10	0.11	0.05	-

^*^p < 0.05.

BMC, bone mineral content; BMD, bone mineral density; DKK1, Dickkopf-related protein 1; OPG, osteoprotegerin; OC, osteocalcin; OPN, osteopontin; SOST, sclerostin; PTH, parathyroid hormone; FGF23, fibroblast growth factor 23, Spearman's rank correlation coefficient was used due to non-normal distribution of biochemical markers.

In the group of patients treated conventionally, a negative correlation was found between FGF23 and BMC (rho = −0.59; *p* < 0.05). In addition, a strong positive correlation was observed between PTH and SOST (rho = 0.61; *p* < 0.05). However, no direct correlations were found between the other markers (DKK1, OPG, OC, and OPN) and bone density indices (Table 6).

**Table 6:** Correlation analysis of bone turnover markers and bone density indices in a group of conventional treatment.

**Parameters**	**L2–L4 BMD**	**L2–L4 T-score**	**Total T-score**	**BMC**	**DKK1**	**OPG**	**OC**	**OPN**	**SOST**	**PTH**	**FGF23**
L2–L4 BMD	-	1.00^*^	0.86^*^	0.36	−0.34	−0.07	0.02	−0.33	−0.11	0.30	−0.15
L2–L4 T-score	1.00^*^	-	0.87^*^	0.36	−0.34	−0.06	0.02	−0.31	−0.12	0.27	−0.18
Total T-score	0.86^*^	0.87^*^	-	0.30	−0.25	−0.16	−0.27	−0.37	−0.17	0.22	−0.21
BMC	0.36	0.36	0.30	-	−0.01	0.16	0.03	0.15	0.48	−0.09	−0.59^*^
DKK1	−0.34	−0.34	−0.25	−0.01	-	0.10	−0.11	0.07	−0.04	0.08	−0.01
OPG	−0.07	−0.06	−0.16	0.16	0.10	-	0.15	0.42	0.11	−0.08	0.24
OC	0.02	0.02	−0.27	0.03	−0.11	0.15	-	0.04	−0.15	0.13	−0.01
OPN	−0.33	−0.31	−0.37	0.15	0.07	0.42	0.04	-	0.05	−0.42	−0.34
SOST	−0.11	−0.12	−0.17	0.48	−0.04	0.11	−0.15	0.05	-	0.61^*^	−0.21
PTH	0.30	0.27	0.22	−0.09	0.08	−0.08	0.13	−0.42	0.61^*^	-	0.31
FGF23	−0.15	−0.18	−0.21	−0.59^*^	−0.01	0.24	−0.01	−0.34	−0.21	0.31	-

^*^p < 0.05.

BMC, bone mineral content; BMD, bone mineral density; DKK1, Dickkopf-related protein 1; OPG, osteoprotegerin; OC, osteocalcin; OPN, osteopontin; SOST, sclerostin; PTH, parathyroid hormone; FGF23, fibroblast growth factor 23, Spearman's rank correlation coefficient was used due to non-normal distribution of biochemical markers.

## 4 Discussion

Chronic inflammation persistent in IBD patients may significantly impact bone turnover. Although it was shown in both CD and UC that bone loss is associated with the action of pro-inflammatory (19–21). However, patients with CD at higher risk of skeletal pathology compared to those with UC (22). Bone marrow TNF-α cells show the ability to promote osteoclastogenesis and excessive bone resorption (23). CX3CL1 expression can promote M1 macrophage polarization and osteoclast differentiation, while inhibition of CX3CL1 expression can reduce inflammation, thereby reducing the predisposition to reduced bone density (24, 25). IBD treatment aims to reduce inflammation, resulting in reduced bone resorption and increased BMD (26, 27). Treatment with anti-TNFα through various mechanisms affects the activity of both osteoclasts and osteoblasts (28).

In our study, BMD (lumbar) and BMC values were similar between the study groups, with the exception of the T-score, which was lower in patients with IBD, especially in CD. Similar results were obtained by Cortés-Berdonces et al. (29) which confirms a certain regularity. However, no differences in BMD and BMC were observed between patients depending on the therapy used, although some data suggest an improvement in BMD after anti-TNFα treatment (30, 31). As shown by Pazianas et al. (31), an increase in BMD was observed mainly with the simultaneous use of infliximab and bisphosphonates, while infliximab treatment alone did not affect BMD.

The most pronounced difference between the groups was elevated OPN levels in patients with CD and UC compared to controls. Although the results of the studies are inconsistent, some authors confirm similar observations (32, 33). Furthermore, Mishima et al. (34) found a positive correlation between serum OPN levels and clinical activity indices in patients with IBD, and therefore propose that OPN be used as a clinical marker of disease activity. OPN reflected inflammation in the colon and rectum, as its plasma levels were reduced and correlations completely disappeared after proctocolectomy (34). In our study, patients were in remission, which may explain the absence of such correlations. However, elevated OPN may reflect increased bone cell activity and constitute a late marker of bone formation, which does not necessarily normalize after anti-TNFα therapy, despite reduced disease activity (35).

With regard to OC, OPG, SOST, PTH, and FGF23, no significant differences were found between patients with CD, UC, and controls, which may indicate a beneficial effect of maintaining remission on bone metabolism parameters. OPG, as an inhibitor of bone resorption by blocking RANKL-RANK interaction (36, 37), should theoretically be modulated by TNF-α and anti-TNFα treatment (38, 39). However, the literature is inconsistent: some studies indicate lower OPG levels (22, 40), others higher in selected groups (41–43). Miheller et al. (44) demonstrated a decrease in OPG after anti-TNFα treatment, whereas in our study, the levels were similar between the groups, with slightly lower values in CD. It is likely that the diagnostic value of OPG may be limited in the assessment of bone metabolic activity in this group of patients

With regard to the Wnt pathway, DKK-1 plays a role in the pathogenesis of CD and bone metabolism (45, 46). The reduction in DKK-1 in pediatric patients treated with anti-TNFα (45) indicates the potential involvement of this pathway in epithelial regeneration and bone remodeling. In our study, DKK-1 levels did not differ significantly between treatment groups, which may reflect the complex regulation of this molecule. The observed positive correlation of DKK-1 with OPG in patients treated with anti-TNFα can be interpreted as a compensatory element in response to chronic inflammation. Similarly, SOST, which acts by inhibiting the Wnt pathway (47), showed no differences between treatment groups, although its values were lower in CD, consistent with other reports (45, 48). Anti-SOST antibody therapy has the potential to restore bone mass (46), but in our study, OC, which is functionally related to osteoblast activity, did not differ significantly between groups and was lower in patients treated with biologics, although the literature indicates a possible increase in OC after anti-TNFα therapy (44, 49).

Furthermore, OC was negatively correlated with SOST in this group of patients, which may suggest that higher SOST activity may be associated with inhibition of osteoblast activity. However, reduced values of these parameters may suggest an imbalance in bone metabolism resulting from chronic inflammation. Marini et al. (50) indicate that treatment with monoclonal antibodies against SOST may promote bone mass restoration, thus preventing bone fractures caused by bone fragility. PTH has been shown to increase calcium reabsorption and inhibit phosphate reabsorption (51).

With regard to PTH, we observed negative correlations between PTH and some DXA parameters, in line with previous reports (52). However, no changes in PTH were found depending on the therapy, which is consistent with some studies (53), although others suggest an increase in OC after anti-TNFα therapy (54). FGF23, synthesized by osteocytes and osteoblasts, may correlate with inflammation and bone turnover (55). Exacerbation of IBD is associated with elevated FGF23 and lower BMD (56). In our study, we observed a negative correlation between FGF23 and BMD in patients treated with anti-TNFα, consistent with previous observations (56). At the same time, its levels did not differ between therapies, and the positive correlation of FGF23 with BMC may indicate secondary, compensatory changes in the hormonal axis (57–59).

The results of our study confirm the presence of bone metabolism abnormalities, which may be present during clinical remission. Therefore, it is worth considering a multimineral supplement for such patients with IBD (60). Biological treatment did not have a clear effect on most of the bone markers we studied, suggesting a varied response to therapies and multifactorial regulation of bone metabolism in IBD. This points to the need for further studies evaluating the long-term effects of chronic inflammation and the treatment used on the skeletal system, taking into account the clinical stage of the disease and the differences between CD and UC. However, individual conclusions cannot be extrapolated to the entire remission.

## 5 Limitations

Our study also has certain limitations. It was a cross-sectional study, and all biochemical and densitometric measurements were taken at a single point in time, during remission of the disease. Therefore, the results obtained reflect the state of bone turnover during maintenance treatment and do not allow for the assessment of changes over time or for conclusions to be drawn about the impact of therapy. In the future, it would also be useful to include information on the individual clinical data of all participants, including detailed disease activity indices (e.g., the Mayo scale) and a complete history of supplementation. The lack of this information may have been an uncontrolled confounding factor affecting the assessed markers of bone turnover. In addition, the study did not measure reference markers of bone turnover, such as P1NP and CTX, due to technical limitations and the availability of laboratory methods. This limits the ability to directly compare the results obtained with other studies using these standard parameters.

## 6 Conclusions

Our results indicate that the choice of anti-TNFα therapy should not be based on the expected differential effects of individual drugs on bone metabolism. Decisions regarding biological therapy should therefore continue to focus primarily on controlling disease activity, while bone status requires parallel and independent assessment and monitoring. In this context, bone turnover markers can serve as complementary tools for identifying patients who require more intensive monitoring or additional supportive interventions to prevent long-term bone complications.

## Data Availability

The raw data supporting the conclusions of this article will be made available by the authors, without undue reservation.

## References

[1] Yu YR Rodriguez JR. Clinical presentation of Crohn's, ulcerative colitis, and indeterminate colitis: symptoms, extraintestinal manifestations, and disease phenotypes. Semin Pediatr Surg. (2017) 26:349–55. doi: 10.1053/j.sempedsurg.2017.10.003 29126502

[2] Liu D Saikam V Skrada KA Merlin D Iyer SS. Inflammatory bowel disease biomarkers. Med Res Rev. (2022) 42:1856–87. doi: 10.1002/med.21893 35603998 PMC10321231

[3] Jeong DY Kim S Son MJ Son CY Kim JY Kronbichler A . Induction and maintenance treatment of inflammatory bowel disease: a comprehensive review. Autoimmun Rev. (2019) 18:439–54. doi: 10.1016/j.autrev.2019.03.002 30844556

[4] Ruemmele F. Consensus guidelines of ECCO/ESPGHAN on the medical management of pediatric Crohn's disease. J Crohns Colitis. (2014) 8:1179–207. doi: 10.1016/S1873-9946(14)50148-1 24909831

[5] Oh HJ Ryu KH Park BJ Yoon BH. Osteoporosis and osteoporotic fractures in gastrointestinal disease. J Bone Metab. (2018) 25:213–7. doi: 10.11005/jbm.2018.25.4.213 30574465 PMC6288610

[6] Lima CA Lyra AC Rocha R Santana GO. Risk factors for osteoporosis in inflammatory bowel disease patients. World J Gastrointest Pathophysiol. (2015) 6:210–8. doi: 10.4291/wjgp.v6.i4.210 26600979 PMC4644885

[7] Kang J Wu X Li Y Zhao S Wang S Yu D. Association between inflammatory bowel disease and osteoporosis in European and East Asian populations: exploring causality, mediation by nutritional status, and shared genetic architecture. Front Immunol. (2024) 15:1425610. doi: 10.3389/fimmu.2024.1425610 39136019 PMC11317921

[8] Hu YQ Jin XJ Lei SF Yu XH Bo L. Inflammatory bowel disease and osteoporosis: common genetic effects, pleiotropy, and causality. Hum Immunol. (2024) 85:110856. doi: 10.1016/j.humimm.2024.110856 39018711

[9] Kawai VK Stein CM Perrien DS Griffin MR. Effects of anti-tumor necrosis factor α agents on bone. Curr Opin Rheumatol. (2012) 24:576–85. doi: 10.1097/BOR.0b013e328356d212 22810364 PMC3753172

[10] Orsolini G Adami G Adami S Viapiana O Idolazzi L Gatti D . Short-term effects of TNF inhibitors on bone turnover markers and bone mineral density in rheumatoid arthritis. Calcif Tissue Int. (2016) 98:580–5. doi: 10.1007/s00223-016-0114-x 26887973

[11] Barnabe C Hanley DA. Effect of tumor necrosis factor alpha inhibition on bone density and turnover markers in patients with rheumatoid arthritis and spondyloarthropathy. Semin Arthritis Rheum. (2009) 39:116–22. doi: 10.1016/j.semarthrit.2008.04.004 18585759

[12] Lichtenstein GR Loftus EV Isaacs KL Regueiro MD Gerson LB Sands BE. ACG clinical guideline: management of Crohn's disease in adults. Am J Gastroenterol. (2018) 113:481–517. doi: 10.1038/ajg.2018.27 29610508

[13] Rubin DT Ananthakrishnan AN Siegel CA Sauer BG Long MD. ACG clinical guideline: ulcerative colitis in adults. Am J Gastroenterol. (2019) 114:384–413. doi: 10.14309/ajg.0000000000000152 30840605

[14] Gomollón F Dignass A Annese V Tilg H Van Assche G Lindsay JO . 3rd European evidence-based consensus on the diagnosis and management of Crohn's disease 2016: part 1: diagnosis and medical management. J Crohns Colitis. (2017) 11:3–25. doi: 10.1093/ecco-jcc/jjw168 27660341

[15] Harbord M Eliakim R Bettenworth D Karmiris K Katsanos K Kopylov U . Third European evidence-based consensus on diagnosis and management of ulcerative colitis. Part 2: current management. J Crohns Colitis. (2017) 11:769–84. doi: 10.1093/ecco-jcc/jjx009. Erratum in: *J Crohns Colitis*. (2017) 11:1512. doi: 10.1093/ecco-jcc/jjx105. Erratum in: *J Crohns Colitis*. (2023) 17:149. doi: 10.1093/ecco-jcc/jjac104 28513805

[16] Krueger D Vallarta-Ast N Checovich M Gemar D Binkley N. BMD measurement and precision: a comparison of GE lunar prodigy and iDXA densitometers. J Clin Densitom. (2012) 15:21–5. doi: 10.1016/j.jocd.2011.08.003 22071029 PMC3336200

[17] Narula N Wong ECL Dulai PS Patel J Marshall JK Yzet C . Defining endoscopic remission in Crohn's disease: MM-SES-CD and SES-CD thresholds associated with low risk of disease progression. Clin Gastroenterol Hepatol. (2024) 22:1687–1696.e6. doi: 10.1016/j.cgh.2024.02.009 38428709

[18] Sharara AI Malaeb M Lenfant M Ferrante M. Assessment of endoscopic disease activity in ulcerative colitis: us simplicity the ultimate sophistication? Inflamm Intest Dis. (2021) 7:7–12. doi: 10.1159/000518131 35224012 PMC8820167

[19] Chedid VG Kane SV. Bone health in patients with inflammatory bowel diseases. J Clin Densitom. (2020) 23:182–9. doi: 10.1016/j.jocd.2019.07.009 31375349

[20] Ghishan FK Kiela PR. Advances in the understanding of mineral and bone metabolism in inflammatory bowel diseases. Am J Physiol Gastrointest Liver Physiol. (2011) 300:G191–201. doi: 10.1152/ajpgi.00496.2010 21088237 PMC3043650

[21] Tilg H Moschen AR Kaser A Pines A Dotan I. Gut, inflammation and osteoporosis: basic and clinical concepts. Gut. (2008) 57:684–94. doi: 10.1136/gut.2006.117382 18408105

[22] Krela-Kazmierczak I Kaczmarek-Ryś M Szymczak A Michalak M Skrzypczak-Zielińska M Drweska-Matelska N . Bone metabolism and the c.-223C > T polymorphism in the 5′UTR region of the osteoprotegerin gene in patients with inflammatory bowel disease. Calcif Tissue Int. (2016) 99:616–24. doi: 10.1007/s00223-016-0192-9 27639566 PMC5097783

[23] Ciucci T Ibáñez L Boucoiran A Birgy-Barelli E Pène J Abou-Ezzi G . Bone marrow Th17 TNFα cells induce osteoclast differentiation, and link bone destruction to IBD. Gut. (2015) 64:1072–81. doi: 10.1136/gutjnl-2014-306947 25298539

[24] Feng X Zhu S Qiao J Ji Z Zhou B Xu W. CX3CL1 promotes M1 macrophage polarization and osteoclast differentiation through NF-κB signaling pathway in ankylosing spondylitis *in vitro*. J Transl Med. (2023) 21:573. doi: 10.1186/s12967-023-04449-0 37626378 PMC10463543

[25] Xiao Q Li X Li Y Wu Z Xu C Chen Z . Biological drug and drug delivery-mediated immunotherapy. Acta Pharm Sin B. (2021) 11:941–60. doi: 10.1016/j.apsb.2020.12.018 33996408 PMC8105778

[26] Guo J Wang F Hu Y Luo Y Wei Y Xu K . Exosome-based bone-targeting drug delivery alleviates impaired osteoblastic bone formation and bone loss in inflammatory bowel diseases. Cell Rep Med. (2023) 4:100881. doi: 10.1016/j.xcrm.2022.100881 36603578 PMC9873828

[27] Bravenboer N Oostlander AE van Bodegraven AA. Bone loss in patients with inflammatory bowel disease: cause, detection and treatment. Curr Opin Gastroenterol. (2021) 37:128–34. doi: 10.1097/MOG.0000000000000710 33332916

[28] Yao Q He L Bao C Yan X Ao J. The role of TNF-α in osteoporosis, bone repair and inflammatory bone diseases: a review. Tissue Cell. (2024) 89:102422. doi: 10.1016/j.tice.2024.102422 39003912

[29] Cortés-Berdonces M Arberas B de la Fuente M Thuissard IJ Marín F. Assessment of bone health in adult patients with inflammatory bowel disease: a single-center cohort study. J Clin Med. (2025) 14:3933. doi: 10.3390/jcm14113933 40507698 PMC12155898

[30] Griffin LM Thayu M Baldassano RN DeBoer MD Zemel BS Denburg MR . Improvements in bone density and structure during anti-TNF-α therapy in pediatric Crohn's disease. J Clin Endocrinol Metab. (2015) 100:2630–9. doi: 10.1210/jc.2014-4152 25919459 PMC4490303

[31] Pazianas M Rhim AD Weinberg AM Su C Lichtenstein GR. The effect of anti-TNF-alpha therapy on spinal bone mineral density in patients with Crohn's disease. Ann N Y Acad Sci. (2006) 1068:543–56. doi: 10.1196/annals.1346.055 16831950

[32] Sato T Nakai T Tamura N Okamoto S Matsuoka K Sakuraba A . Osteopontin/Eta-1 upregulated in Crohn's disease regulates the Th1 immune response. Gut. (2005) 54:1254–62. doi: 10.1136/gut.2004.048298 16099792 PMC1774642

[33] Komine-Aizawa S Masuda H Mazaki T Shiono M Hayakawa S Takayama T. Plasma osteopontin predicts inflammatory bowel disease activities. Int Surg. (2015) 100:38–43. doi: 10.9738/INTSURG-D-13-00160.1 25594638 PMC4301292

[34] Mishima R Takeshima F Sawai T Ohba K Ohnita K Isomoto H . High plasma osteopontin levels in patients with inflammatory bowel disease. J Clin Gastroenterol. (2007) 41:167–72. doi: 10.1097/MCG.0b013e31802d6268 17245215

[35] Choi ST Kim JH Kang EJ Lee SW Park MC Park YB . Osteopontin might be involved in bone remodelling rather than in inflammation in ankylosing spondylitis. Rheumatology (Oxford). (2008) 47:1775–9. doi: 10.1093/rheumatology/ken385 18854347

[36] Udagawa N Koide M Nakamura M Nakamichi Y Yamashita T Uehara S . Osteoclast differentiation by RANKL and OPG signaling pathways. J Bone Miner Metab. (2021) 39:19–26. doi: 10.1007/s00774-020-01162-6 33079279

[37] Nahidi L Leach ST Lemberg DA Day AS. Osteoprotegerin exerts its pro-inflammatory effects through nuclear factor-κB activation. Dig Dis Sci. (2013) 58:3144–55. doi: 10.1007/s10620-013-2851-2 24048682

[38] Yao Z Getting SJ Locke IC. Regulation of TNF-induced osteoclast differentiation. Cells. (2021) 11:132. doi: 10.3390/cells11010132 35011694 PMC8750957

[39] Saidenberg-Kermanac'h N Corrado A Lemeiter D deVernejoul MC Boissier MC Cohen-Solal ME. TNF-alpha antibodies and osteoprotegerin decrease systemic bone loss associated with inflammation through distinct mechanisms in collagen-induced arthritis. Bone. (2004) 35:1200–1207. doi: 10.1016/j.bone.2004.07.004 15542046

[40] Priadko K Moretti A Iolascon G Gravina AG Miranda A Sgambato D . Bone alterations in inflammatory bowel diseases: role of osteoprotegerin. J Clin Med. (2022) 11:1840. doi: 10.3390/jcm11071840 35407448 PMC8999800

[41] Bernstein CN Sargent M Leslie WD. Serum osteoprotegerin is increased in Crohn's disease: a population-based case control study. Inflamm Bowel Dis. (2005) 11:325–30. doi: 10.1097/01.MIB.0000164015.60795.ca 15803021

[42] Krela-Kazmierczak I Szymczak-Tomczak A Łykowska-Szuber L Wysocka E Michalak M Stawczyk-Eder K . Interleukin 6, osteoprotegerin, sRANKL and bone metabolism in inflammatory bowel diseases. Adv Clin Exp Med. (2018) 27:449–53. doi: 10.17219/acem/75675 29558031

[43] Moschen AR Kaser A Enrich B Ludwiczek O Gabriel M Obrist P . The RANKL/OPG system is activated in inflammatory bowel disease and relates to the state of bone loss. Gut. (2005) 54:479–87. doi: 10.1136/gut.2004.044370 15753532 PMC1774465

[44] Miheller P Muzes G Rácz K Blázovits A Lakatos P Herszényi L . Changes of OPG and RANKL concentrations in Crohn's disease after infliximab therapy. Inflamm Bowel Dis. (2007) 13:1379–84. doi: 10.1002/ibd.20234 17663430

[45] Kim MJ Choe YH. Correlation of Dickkopf-1 with inflammation in Crohn disease. Indian Pediatr. (2019) 56:929–32. doi: 10.1007/s13312-019-1649-5 31729323

[46] Palatianou ME Karamanolis G Tsentidis C Gourgiotis D Papaconstantinou I Vezakis A . Signaling pathways associated with bone loss in inflammatory bowel disease. Ann Gastroenterol. (2023) 36:132–40. doi: 10.20524/aog.2023.0785 36864939 PMC9932862

[47] Jia YK Yu Y Guan L. Corrigendum: Advances in understanding the regulation of pluripotency fate transition in embryonic stem cells. Front Cell Dev Biol. (2024) 12:1523967. doi: 10.3389/fcell.2024.1523967 39620141 PMC11605082

[48] Wu X Ai Y He Y Ma D Li X Huang X . Localized sclerostin accumulation in osteocyte lacunar-canalicular system is associated with cortical bone microstructural alterations and bone fragility in db/db male mice. Front Cell Dev Biol. (2025) 13:1562764. doi: 10.3389/fcell.2025.1562764 40352664 PMC12062167

[49] Veerappan SG Healy M Walsh B O'Morain CA Daly JS Ryan BM. A 1-year prospective study of the effect of infliximab on bone metabolism in inflammatory bowel disease patients. Eur J Gastroenterol Hepatol. (2016) 28:1335–44. doi: 10.1097/MEG.0000000000000719 27508327

[50] Marini F Giusti F Palmini G Brandi ML. Role of Wnt signaling and sclerostin in bone and as therapeutic targets in skeletal disorders. Osteoporos Int. (2023) 34:213–38. doi: 10.1007/s00198-022-06523-7 35982318

[51] Leung EKY. Parathyroid hormone. Adv Clin Chem. (2021) 101:41–93. doi: 10.1016/bs.acc.2020.06.005 33706890

[52] Qu Z Yang F Hong J Wang W Yan S. Parathyroid hormone and bone mineral density: a Mendelian randomization study. J Clin Endocrinol Metab. (2020) 105:dgaa579. doi: 10.1210/clinem/dgaa579 32827441

[53] Szentpétery Á Horváth Á Gulyás K Pethö Z Bhattoa HP Szántó S . Effects of targeted therapies on the bone in arthritides. Autoimmun Rev. (2017) 16:313–20. doi: 10.1016/j.autrev.2017.01.014 28159704

[54] Gulyás K Horváth Á Végh E Pusztai A Szentpétery Á Pethö Z . Effects of 1-year anti-TNF-α therapies on bone mineral density and bone biomarkers in rheumatoid arthritis and ankylosing spondylitis. Clin Rheumatol. (2020) 39:167–75. doi: 10.1007/s10067-019-04771-3 31522318

[55] David V Francis C Babitt JL. Ironing out the cross talk between FGF23 and inflammation. Am J Physiol Renal Physiol. (2017) 312:F1–8. doi: 10.1152/ajprenal.00359.2016 27582104 PMC5283889

[56] El-Hodhod MA Hamdy AM Abbas AA Moftah SG Ramadan AA. Fibroblast growth factor 23 contributes to diminished bone mineral density in childhood inflammatory bowel disease. BMC Gastroenterol. (2012) 12:44. doi: 10.1186/1471-230X-12-44 22551310 PMC3438067

[57] Oikonomou KA Orfanidou TI Vlychou MK Kapsoritakis AN Tsezou A Malizos KN . Lower fibroblast growth factor 23 levels in young adults with Crohn disease as a possible secondary compensatory effect on the disturbance of bone and mineral metabolism. J Clin Densitom. (2014) 17:177–84. doi: 10.1016/j.jocd.2013.03.019 23623649

[58] Jurina A Kasumović D Delimar V Filipec KaniŽaj T Japjec M Dujmović T . Fibroblast growth factor 23 and its role in bone diseases. Growth Factors. (2024) 42:1–12. doi: 10.1080/08977194.2023.2274579 37906060

[59] Sirikul W Siri-Angkul N Chattipakorn N Chattipakorn SC. Fibroblast growth factor 23 and osteoporosis: evidence from bench to bedside. Int J Mol Sci. (2022) 23:2500. doi: 10.3390/ijms23052500 35269640 PMC8909928

[60] Aslam MN Turgeon DK Appelman HD Stidham R McClintock S Allen R . multi-mineral intervention to improve disease-related and mechanistic biomarkers in ulcerative colitis patients: results from a randomized trial. PLoS One. (2025) 20:e0337408. doi: 10.1371/journal.pone.0337408 41359652 PMC12685183

