# Supplementary material for: Bone turnover markers in patients with inflammatory bowel disease in remission: a cross-sectional comparison of anti-TNFα therapy with conventional maintenance therapy
Source: Front Med (Lausanne). 2026 Feb 10;13:1729584. doi: 10.3389/fmed.2026.1729584 (PMC12929429; doi:10.3389/fmed.2026.1729584)
Supplement: Supplementary file 1 [file Supplementary_file_1.docx]

Supplementary Material

Table 1. Characteristics of the study population

| Variables | | CD group (n=35) | UC group (n=37) | Control group (n=28) | p-value |
| --- | --- | --- | --- | --- | --- |
| Age (year) | | 34.9 ± 12.04 | 36.9 ± 11.17 | 34.7 ± 10.11 | .707 |
| Height (cm) | | 172.3 ± 9.43 | 170.9 ± 7.68 | 170.6 ± 11.05 | .725 |
| Weight (kg) | | 69.9 ± 14.84 | 74.2 ± 25.50 | 73.4 ± 12.76 | .597 |
| BMI (kg/m2) | | 23.7 ± 3.88 | 24.3 ± 5.35 | 25.4 ± 3.30 | .259 |
| Sex | Female | 12 (34.3%) | 18 (48.6%) | 19 (67.9%) | χ2=7.02  **.030** |
|  | Male | 23 (65.7%) | 19 (51.4%) | 9 (32.1%) |  |
| Treatment | Anti-TNFα | 28 (80.0%) | 27 (73.0%) | N/A | χ2=0.49  .483 |
|  | Conventional | 7 (20.0%) | 10 (27.0%) |  |  |

Abbreviations: BMI – Body Mass Index, CD – Crohn's disease, UC – ulcerative colitis.

^a Comparison between CD and UC.^

Table 2. Comparison of hemoglobin, c-reactive protein, and calprotectin values

| Study group | Variables | N | M | Me | SD |
| --- | --- | --- | --- | --- | --- |
| CD | CRP | 33 | 4.14 | 3.1 | 3.38 |
|  | Hemoglobin (g/dl) | 33 | 13.59 | 13.6 | 1.66 |
|  | Calprotectin (ug/g) | 24 | 49.74 | 39.6 | 31.91 |
| UC | CRP | 34 | 2.22 | 1.75 | 1.68 |
|  | Hemoglobin (g/dl) | 35 | 13.22 | 10.5 | 1.19 |
|  | Calprotectin (ug/g) | 21 | 27.9 | 5.0 | 23.63 |

CD – Crohn's disease, UC – ulcerative colitis, M – mean, Me – median, SD – standard deviation, CRP – C-reactive protein
